# Proteomic Profiling in Multiple Sclerosis Clinical Courses Reveals Potential Biomarkers of Neurodegeneration

**DOI:** 10.1371/journal.pone.0103984

**Published:** 2014-08-06

**Authors:** Maria Liguori, Antonio Qualtieri, Carla Tortorella, Vita Direnzo, Angelo Bagalà, Mariangela Mastrapasqua, Patrizia Spadafora, Maria Trojano

**Affiliations:** 1 National Research Council of Italy, Institute for Biomedical Technologies, Bari, Italy; 2 National Research Council of Italy, Institute of Neurological Sciences, Mangone (CS), Italy; 3 Department of Basic Medical Sciences, Neurosciences and Sense Organs, University of Bari, Bari, Italy; Innsbruck Medical University, Austria

## Abstract

The aim of our project was to perform an exploratory analysis of the cerebrospinal fluid (CSF) proteomic profiles of Multiple Sclerosis (MS) patients, collected in different phases of their clinical course, in order to investigate the existence of peculiar profiles characterizing the different MS phenotypes. The study was carried out on 24 Clinically Isolated Syndrome (CIS), 16 Relapsing Remitting (RR) MS, 11 Progressive (Pr) MS patients. The CSF samples were analysed using the Matrix Assisted Laser Desorption Ionisation Time Of Flight (MALDI-TOF) mass spectrometer in linear mode geometry and in delayed extraction mode (*m/z* range: 1000–25000 Da). Peak lists were imported for normalization and statistical analysis. CSF data were correlated with demographic, clinical and MRI parameters. The evaluation of MALDI-TOF spectra revealed 348 peak signals with relative intensity ≥1% in the study range. The peak intensity of the signals corresponding to Secretogranin II and Protein 7B2 were significantly upregulated in RRMS patients compared to PrMS (p<0.05), whereas the signals of Fibrinogen and Fibrinopeptide A were significantly downregulated in CIS compared to PrMS patients (p<0.04). Additionally, the intensity of the Tymosin β4 peak was the only signal to be significantly discriminated between the CIS and RRMS patients (p = 0.013). Although with caution due to the relatively small size of the study populations, and considering that not all the findings remained significant after adjustment for multiple comparisons, in our opinion this mass spectrometry evaluation confirms that this technique may provide useful and important information to improve our understanding of the complex pathogenesis of MS.

## Introduction

One of the most challenging aspects in Multiple Sclerosis (MS) research is the search for prognostic markers that can help clinicians in their decision-making plans for treatment. Currently, the common strategy is to start disease-modifying drugs (DMD) at the earliest stages of this disease in order to delay the conversion of Clinically Isolated Syndrome (CIS) into Clinically Definite MS (CDMS) [1]–[3], and the shift from the relapsing course MS (RRMS) into the secondary progression MS (SPMS). Demographic and clinical variables, such as an older age at onset, male gender and high early relapse rate, together with lesion load and other MRI metrics [4], and recent evidence of activated cellular pathways in the peripheral immune system [5], have been ascribed as significant determinants in the rapid transition to progressive MS deterioration.

The search for cerebrospinal fluid (CSF) immunological and/or neurodegenerative biomarkers by means of proteomic profiling may provide important information about the pathogenic processes underlying the disease progression. Moreover, recent improvements in proteomic approaches by mass spectrometry represent a step forward in the direction of identifying useful predictive biomarkers. Schultzer et al. reported significant differences in several CSF proteins related to the cerebral grey matter (*i.e.* Nogo receptors) between CIS and relapsing CDMS [6].

Mass spectrometry has previously been demonstrated to improve the accuracy of diagnosis in MS-related disorders such as NMO [7], and was used to generate important data on factors that predict the conversion from CIS to CDMS in pediatric MS [8]. Moreover, Matrix Assisted Laser Desorption Ionisation Time Of Flight (MALDI-TOF) mass spectrometry appears to be a more suitable tool in the search for peptides and small proteins differentially expressed in CSF [9]. This is because MALDI-TOF allows the measurement of the intensities of endogenous peptides produced by fibrinolysis (coagulation factors) or immune response (complement fractions), or inflammation (β_2_-microglobulin) or neurosecretion (chromogranines).

To date, several results have been obtained from the CSF proteome in the >20000 Dalton range. Our purpose is to search for peptides and small proteins differentially expressed in the restricted, but very important, CSF peptidome range <15000 Daltons in a population of patients with different MS phenotypes by using direct MALDI-TOF mass spectroscopy profiling. Data derived from this analysis could provide additional information on the pathogenic aspects of MS during the crucial steps of the disease and facilitates the detection of markers of disease progression.

## Materials and Methods

### Ethic Statement

The approval for the conduct of this study was obtained from the ethical committee of the “Azienda Sanitaria Provinciale Cosenza, Italy”, and of the “Azienda Consorziale Policlinico, University of Bari, Italy”, in agreement with the Declaration of Helsinki.

The written informed consent was obtained from all patients and controls under the protocol approved by the Institutions’ review boards.

### Subjects

The study population included 24 CIS, 16 RRMS, 11 Progressive (Pr) MS patients [10]–[12] who were followed by neurologists with a primary interest in MS at the Neurophysiopathology Unit (NPU), Department of Basic Medical Science, Neuroscience and Sense Organs, University of Bari (Italy). At the time of the lumbar puncture, 8 out of 24 CIS and 4 out of 16 RRMS were in the relapsing phase of the disease, a clinical relapse being defined as new or recurrent neurologic symptoms not associated with fever or infection, lasting at least 24 hours [13]. They all started the steroid treatments after the CSF sampling, and the minimum time lag between the lumbar puncture and any previous steroid administration was 30 days. Furthermore, none of the MS patients was under DMD therapy at that time.

CSF samples were collected in a period of time ranging from 2005 to 2012; they were treated with protease inhibitors and stored at −80°C within the subsequent 2 hours, according to a consensus protocol [14]. All MS patients were followed for at least 2 years at the NPU where they underwent clinical and MRI examinations every 6 months. Clinical (relapses and EDSS) [15] and radiological (T2 lesion load, Gd-enhancement) data were collected from the iMed database [16].

In order to verify the pathological specificity of the significant peak signals, if any, emerged from the analysis [17], 18 patients with other neurological diseases (OND) were included in the study; 7 patients had a primary cognitive impairment, from now on termed “dementia” and 11 were diagnosed with Chronic Inflammatory Demyelinating Polyneuropathy, CIDP) (**Table 1** for details).

**Table 1:** Comparisons of clinical and demographic features among the MS and OND subgroups.

	CIS (n. 24)	RRMS (n. 16)	PrMS (n. 11)	dementia (n. 7)	CIDP (n. 11)
female/male°	18/6	12/4	5/6	4/3	0/11
mean age at onset (SD) years*	30.8 (8.4)	26.9 (9.6)	39.4 (16.2)	60.6 (9.7)	52.8 (17.2)
mean disease duration at LP (SD) months∧	5.0 (6.2)	16.0 (27.9)	176.6 (157.6)	23.2 (15.6)	62.8 (92.0)
mean EDSS baseline (SD)	1.9 (1.0)	2.0 (0.7)	4.5 (0.9)	–	–
OB (mean no, SD)	13.8 (7.7)	12.5 (7.3)	11.3 (8.2)	–	–

**Footnote:** LP = Lumbar Puncture; OB = oligoclonal bands.

*Fisher’s exact test:* °p = 0.0004*;*

*Kruskall-Wallis test:* *Age at onset: p = 0.0001; ∧Disease duration at LP: p = 0.0001.

### MRI protocol

MRI examinations of the MS subjects were performed at the Department of Basic Medical Science, Neuroscience and Sense Organs, University of Bari (Italy) by using a 1.5T scanner. MS patients underwent conventional MRI scans within 26.3+/−57.6 days from the CSF sample; the sessions consisted of:

1. axial T2-weighted fast spin echo (FSE) (repetition time (TR)/echo time (TE):4850/81 msec; field of view (FOV): 250×250; matrix size 256×256; 42 contiguous slices; slice thickness: 3 mm);
2. axial fluid-attenuated inversion recov­ery (FLAIR) (TR/TE:11000/130 msec; inversion time (TI): 2250; FOV: 250×250; matrix size 256×256; 24 contiguous slices; slice thickness: 3 mm);
3. axial fast spoiled gradient echo (FSPGR)T1-weighted (TR/TE:275/msec; FOV: 250×250; matrix size 256×256; 24 contiguous slices; slice thickness: 3 mm)
4. postcontrast T1-weighted sequence with the same parameters as the precontrast T1-weighted scans, five minutes after Gadolinium i.v. administration.

### Sample preparation for MALDI-TOF MS profiling

The CSF protein concentration was measured using the Bio-Rad Protein Assay (Bio-Rad Laboratories, Hercules, CA - USA) according to the manufacturer’s instructions. An aliquot of each CSF sample, containing approximately 5 milligrams (mg) of protein, was subjected to a desalting/concentration step over a Zip Tip C18 (Millipore Corporation, Billerica MA - USA) using 50% acetonitrile/TFA 0.1% as elution buffer. The eluted samples were typically mixed at a 1∶1 v/v ratio with a α-cyano-4-hydroxycinnamic acid (CHCA) matrix solution (5 mg/mL in 50% ACN and 0.1% TFA), and 1 milliliter (mL) of this solution was deposited onto stainless steel target surfaces and allowed to dry at room temperature. Peptide/protein profiles were analysed using a Voyager DE PRO MALDI-TOF mass spectrometer (AB Sciex, Framingham, MA - USA) equipped with a 337 nm nitrogen laser and delayed-extraction (DE) technology. Separate spectra were obtained for a restricted mass-to charge (*m/z*) range (1000–25000 Da) in linear mode geometry by applying an acceleration voltage of 25 kV, and the DE was maintained at 250 ns to give an appropriate time-lag focus after each laser shot. Each individual spectrum, representing ten accumulated subspectra, was obtained using 1000 laser shots. The acquired raw spectra were then processed for automated advanced baseline correction and noise filter with the Voyager Data Explorer software, version 4.1 (ABSciex, Framingham, MA - USA). Finally, the peak lists were imported for data transformation and statistical analysis. A normal reference mass list was used to compare the patient’s spectra as previously published [18]. The MS spectra were also evaluated with great accuracy to reveal the presence of hemoglobin chains at 15.127+/−0.5 (alpha) and 15.868+/−0.5 (beta) m/z. Each sample was assayed in duplicate; intra- and inter- experimental variations for this procedure were previously tested [18].

### Statistical analysis

The influence of mass spectrometry noise signals was reduced by collecting and comparing only data signals with a relative intensity equal or superior to 1%. The peak area of each signal was normalized as a percentage of the total peak area (individual peak area/total peak area percent) [19]. All values were expressed as means +/− SD and the statistical significance was set at p≤0.05).

Kruskal-Wallis and Fisher’s exact tests were applied in order to compare clinical and demographic features among the MS and OND subgroups.

Following the exploratory nature of this investigation, a hierarchical cluster analysis using Ward’s method and applying squared Euclidean distance was applied in order to discriminate between the overall peptide signals and to obtain homogeneous classes (clusters) of MS subjects by smaller numbers of peak signals.

The relative peak areas of the selected signals, resulting from the previous analysis, were then analyzed among MS and OND patients by applying the Kruskal-Wallis test (among all MS groups) and the Mann-Whitney U-test (between groups), in the absence of a normal distribution of data in the analysed subgroups.

Given the exploratory nature of this investigation and in order to minimize type II errors, we decided to present the results of the single comparisons without the adjustment for multiple testing [20], [21]. However, for the completeness of the analysis some multiple hypothesis corrections were also addressed by controlling the Benjamini-Hochberg False Discovery Rate associated to each p-value [22].

The Spearman’s rank correlation test was performed using the demographic and clinical features of MS patients, and the intensity of the peak signals of interest, with the Statistical Package for Social Science software, SPSS version 18.0, Chicago, IL - USA.

## Results

Clinical and demographic features of the MS and OND patients are summarized in **Table 1**; significant differences were found for age at disease onset among all the study groups (p = 0.0001), whereas both age at onset (p = 0.048) and baseline disability score (p = 0.0001) significantly differed among the MS subgroups.

The analysis of the MALDI-TOF spectra revealed 348 peak signals with relative intensity ≥1% in the m/z range below 15000 Da. Among the MS groups, CIS differed from RRMS patients for the intensity of 17 signals and from PrMS for 35 peak signals. The MS population significantly differed from OND for the intensity of 75 peak signals (**Table S1 in Appendix S1**).

**Figure 1:**
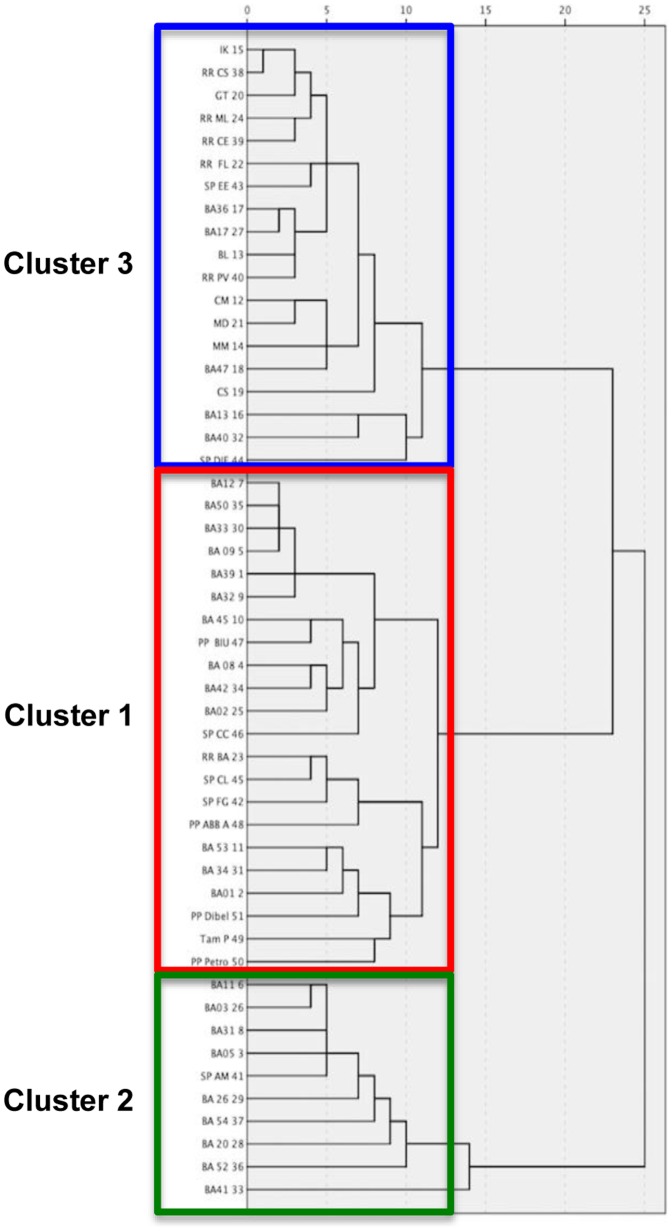
Dendrogram that resulted from the clusters analysis within the MS population. See **Table 2** for details.

Hierarchical cluster analysis (partial views in **Figure 1**) returned 3 groups of MS patients who showed 153 differentially expressed signals (p<0.05); among them we focused on peak signals already identified from previous studies published in the literature and corresponding to proteins of interest (**Table 2**, complete list in **Table S2 in Appendix S1**). The clusters also differed for the age at disease onset that was significantly older for patients enclosed in cluster-1 (p = 0.07 *versus* cluster-2, and p = 0.034 *versus* cluster-3). We also noted that cluster-1 was represented more frequently in the PrMS course than in the other two courses, whereas CIS subjects were more often grouped in cluster 3. However, these data did not reach statistical significance (p = 0.06) (**Table 2**).

**Table 2:** Clinical differences between clusters, and list of defined peptides that resulted differentially expressed between the subgroups.

	Accession Number	Cluster 1 (no.22)	Cluster 2 (no.10)	Cluster 3 (no.19)
MS course (CIS/RR/Pr)	–	9/5/8	3/6/1	12/5/2
Age at disease onset (years)	–	36.32 (11.93)	23.191 (9.26)	29.81 (9.78)
Complement 3F (1305–1319)	P01024	0.18 (0.22)	0.04 (0.04)	0.05 (0.13)
Secretogranin II (529–566)	P13521	2.29 (1.05)	3.97 (1.68)	2.31 (0.77)
Protein 7B2 (182–212)	P05408	4.63 (1.37)	8.87 (2.82)	6.74 (1.37)
Chromogranin A (97–131)	P10645	2.99 (1.35)	0.74 (1.11)	3.43 (0.73)
Chromogranin B (273–303)	P05060	0.22 (0.23)	0.031 (0.08)	0.73 (0.37)
Thymosin β4	P62328	0.23 (0.24)	0.021 (0.05)	0.36 (0.29)
Osteopontin (249–314)*	P10451	0.07 (0.10)	0.003 (0.02)	0.23 (0.11)
Fibrinogen (560–600)	P02671	0.17 (0.29)	0.11 (0.15)	0.13 (0.45)
Fibrinopeptide A (21–35)	P02671	0.37 (0.42)	0.55 (0.75)	0.95 (2.45)

**Footnote:** *Peptide phosphorylated.

**Figure 2:**
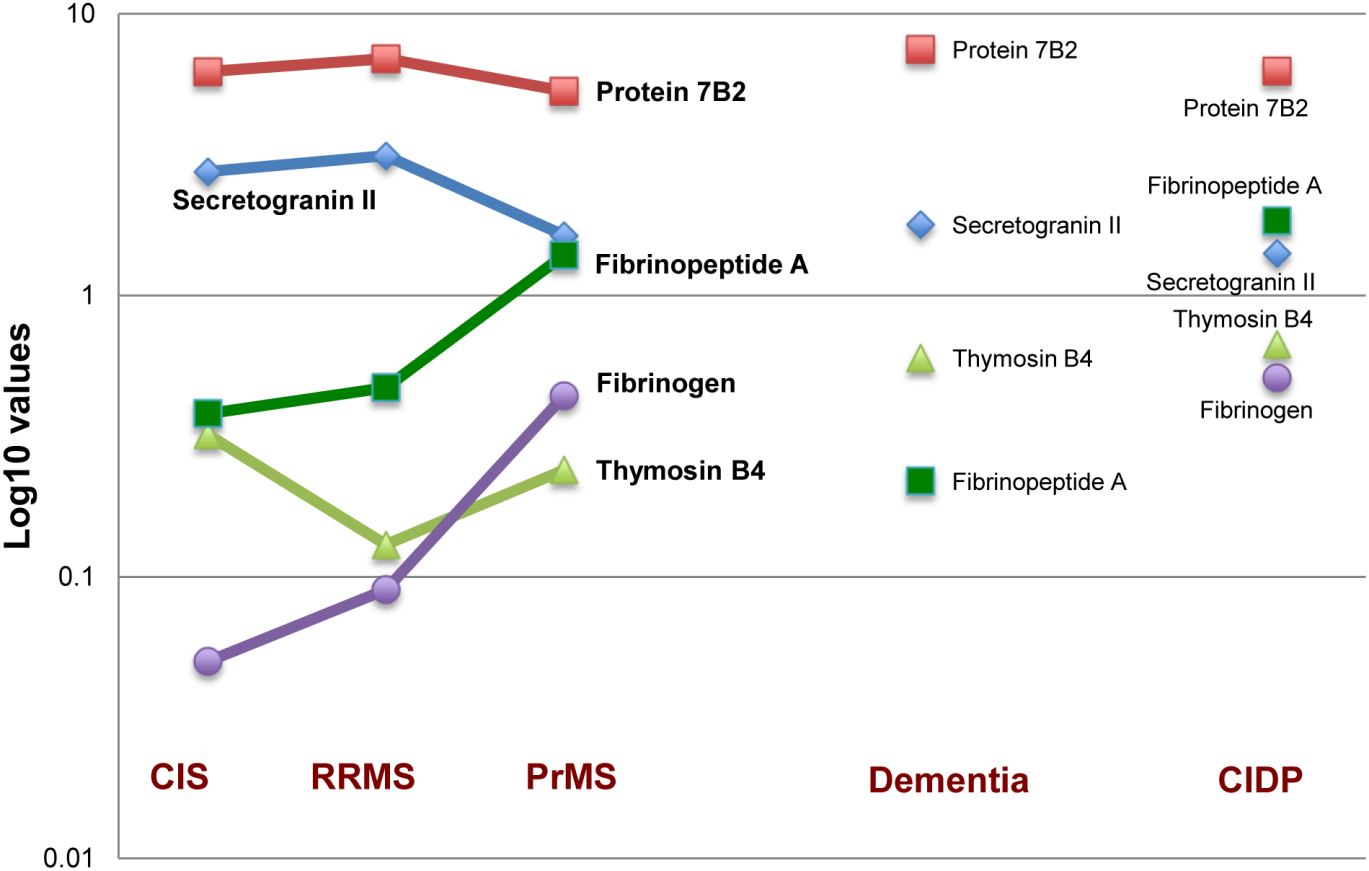
Distribution of selected peak signals intensities in the different MS groups and in dementia and CIDP patients. Values on Y-axes are expressed as log10 of peak signals intensity; *Fibrinogen signal is not detectable in patients with dementia.

We examined these proteins in order to identify possibly distinctive patterns within the different CIS/MS subgroups (**Figure 2** **,** **Table 3**).

**Table 3:** Fold-changes of the peak signals intensity between the subgroups.

Protein Name	PrMS *versus* CIS	PrMS *versus* RRMS	CIS *versus* RRMS	MS *versus* dementia	MS *versus* CIDP
Protein 7B2	−1.2	−1.3 (0.05)	−1.1	−1.2	1.0
Secretogranin II	−1.7 (0.001)∧	−1.9 (0.007)	−1.1	1.5	1.9 (0.001)∧
Thymosin β4	−1.3	1.8	2.5 (0.013)	−2.5 (0.043)	−2.8
Fibrinogen	8.8 (0.005)	4.9 (0.05)	−1.8	*NA	−3.4
Fibrinopeptide A	3.7 (0.04)	3	−1.2	2.8	−3.0 (0.039)

**Footnote:** Fold changes have been calculated as means of subgroups intensity ratios (in parenthesis p-values<0.05).

*the peak intensity of Fibrinogen signal is not detectable in patients with dementia.

∧p = 0.032 after False Discovery Rate adjustment.

Secretogranin II (ScgII) showed a significant higher intensity in CIS and RRMS patients than in PrMS patients (p<0.007), whereas Protein 7B2 (P7B2) was found significantly higher only in RRMS *versus* PrMS patients (p = 0.05). Thymosin β4 (Tβ4) was significantly upregulated in CIS than in RRMS patients (p = 0.013), whereas it was significantly downregulated in the whole MS population compared to the patients with dementia (p = 0.043). Finally, the peak signal corresponding to Fibrinogen (Fbg) was found significantly upregulated in PrMS compared to CIS (p = 0.005) and RRMS (p = 0.047) subjects, and the intensity of the signal related to Fibrinopeptide A (FPA) was significantly higher in PrMS compared to CIS subjects (p = 0.040) (**Table 3**). Interestingly, False Discovery Rate adjustment confirmed the significant upregulation of ScgII in CIS *versus* PrMS patients, and in the overall MS population *versus* CIDP subjects (p<0.031).

The remaining peak signals that resulted differentially expressed in the cluster analysis are still under investigation for their identification. Among them, we observed a signal at 3817.45 *m/z* whose intensity varied extremely especially within the CIS subjects (from 0.00 to 19% - **Figure S1 in Appendix S1**). This signal showed significant high intensity in the (small, only 5 patients) PPMS group (range: 0.00–10.45%), whereas it was very low in OND patients (range: 0.00–1.87%). Only in the CIS group, this signal significantly correlated with Gd-enhancement at baseline MRI (p = 0.046).

At the end of the 2-years follow-up, 19/24 CIS patients had converted to CDMS; no baseline clinical and demographic variables discriminated the subgroup of patients who had converted to CDMS but the two subgroups differed in 8 peak signals showing significant different intensities, although they did not survive the False Discovery Rate adjustment (**Table S3 in Appendix S1**).

### Correlations between peak signals intensities and clinical features in the study population

**Figure 3:**
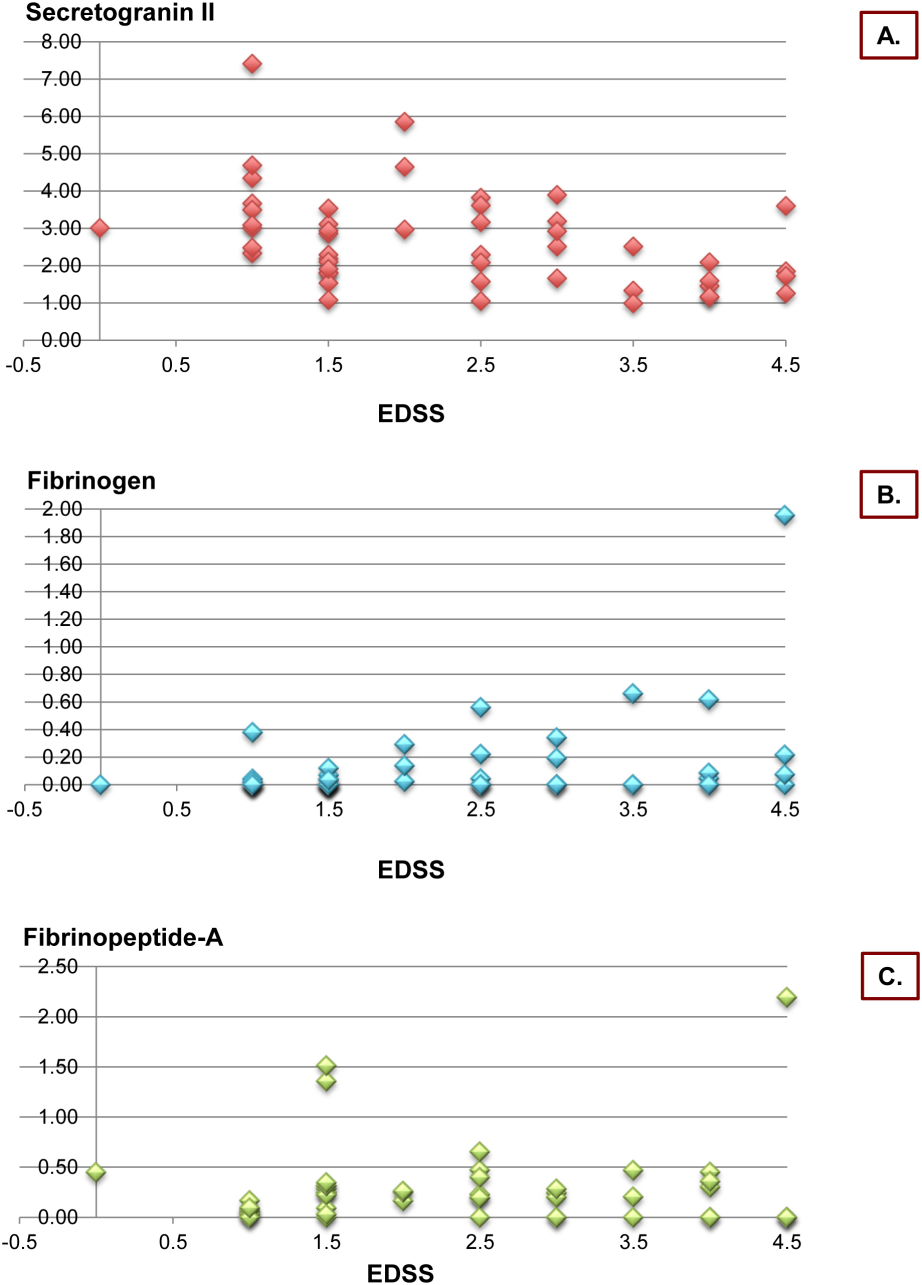
Scattered plots representing the correlations of (A) Secretogranin II, (B) Fibrinogen, (C) Fibrinopeptide-A, and the EDSS scores in the MS population.

FPA significantly correlated with the EDSS score at the study entry (correlation coefficient, cc = 0.4, p = 0.012) (Figure 3) and the number of CSF oligoclonal bands (cc = −0.4, p = 0.003) (**Figure 3**).

The intensity of the Fbg signal was only found to be significantly correlated with the number of Functional Systems (FS) involved at the disease onset (cc = 0.5, p = 0.001) and the baseline EDSS score (cc = 0.4, p = 0.007) (**Figure 3**).

P7B2 and ScgII were inversely correlated with the age at disease onset (cc = −0.4, p = 0.002 and cc = −0.4, p = 0.001, respectively); ScgII also correlated with the number of FS at onset (cc = −0.4, p = 0.001) and the basal EDSS score (cc = −0.5, p<0.001) (**Figure 3**).

## Discussion

To our knowledge, this is one of the very few studies that sought to identify *native* (not pre-treated or digested) small proteins or peptides differentially expressed in MS by using direct MALDI-TOF mass spectroscopy profiling. Our analysis, although exploratory and performed in a relatively small sample, revealed several peak signals whose intensities seemed to discriminate between the different subgroups. The overall trend showed that ScgII was upregulated in CIS and RRMS patients compared to PrMS patients, whereas the Fbg signal was downregulated in the same subgroups. Additionally, the intensity of Tβ4 peak was the only significantly discriminating signal between the CIS and RRMS patients.

MS is a chronic autoimmune disease of the central nervous system in which both the inflammatory demyelination and the axonal injury contribute the phenotypes. Since it is characterized by significant heterogeneity of clinical and radiological patterns [23], [24], the ability to identify true predictive markers has so far been elusive [25]. One possible reason for this may be because of the multi-factorial nature of MS that involves several genes and their interactions [26], [27], as well as the intervention of environmental factors [28], [29] that leave many MS pathogenic issues still unsolved.

Proteomic analyses of different human compartments by using sensitive approaches like mass spectroscopy have generated data on the pathological features of MS, despite the limitation of being a time-consuming technique [30]. Stoop et al [31] evaluated the CSF proteomic profiles of RR/PP MS patients by MALDI-FTICR (Fourier Transform Ion Cyclotron Resonance) mass spectrometry and confirmed the role of proteins related to Vitamin D homeostasis. On the other hand, a chemical labelling approach in combination with Liquid Chromatography-Electrospray Ionization mass spectrometry revealed that the Chitinase 3-like 1 protein was associated with the conversion from CIS to CDMS [32].

In the present investigation, for each MS subgroups we generated proteomic profiles that frequently overlap with each other, as reported by others [31]. For this reason we were not able to identify predictive markers of MS progression, for example from CIS to CDMS, although we are still working on the characterization of those peak signals that were significantly different between the two groups. However, we believe that our results may have provided further insights into the function of several proteins in MS.

In our opinion, one of the most interesting finding derived from the Granins group: ScgII and P7B2 (also known as ScgV). The potential role of these proteins in MS and other neurodegenerative diseases has been recently stated.

SgcII, identified in both neurons and glia, was found to exert cellular chemoattractive effect on monocytes, eosinophils and endothelial cells during inflammation, and it was able to influence the neurite outgrowth [33]. Mattsson et al [34], [35] showed lower SgcII levels in a mixed MS population compared to healthy controls (HC) and Alzheimer’s Disease (AD) subjects, and a significant correlation between these levels and AβPP-derived peptides, suggesting that it might be involved in the cerebral neurodegeneration connected to amyloid metabolism [35].

In our study, the ScgII peak signal intensity was higher in CIS and RRMS patients than in PrMS and CIDP patients; with interest, some of them remained significant after the adjustment for multiple testing (CIS *versus* PrMS). In our view, this evidence is consistent with acute inflammation and the spared neuronal regenerative abilities in the earliest MS phases whereas, as the disease progresses, the significant downregulation of the protein leads to axonal loss and cellular degeneration. In line with this hypothesis, SgcII levels were correlated with lower EDSS scores at the study entry and fewer numbers of FSs involved at the onset.

Consistent with this data was the observation that the peak intensity of P7B2 was higher in RRMS patients when compared to PrMS, although this result did not survive the adjustment for multiple comparisons. Interestingly, this neural protein has been described to decrease the folding of Aβ proteins, whereas its loss appeared to induce *in vitro* Aβ1-42 neurotoxicity, which showed a novel anti-aggregational role in neurodegenerative diseases such as Alzheimer’s and Parkinson’s Diseases [36]. Since the function of Aβ1-42 has been evoked in MS pathogenesis without definitive results [37], [38], it is reasonable to hypothesize that the P7B2 upregulation observed during the earliest (and potentially more inflammatory) MS courses might represent a cellular protection from the neurotoxicity of Aβ proteins, and that this function is reduced during the disease progression consistent with the decreased protein signal.

Other suggestive information has been derived from the FPA and Fbg signals. Recently, the implication of Fbg and its fragments which leaked from the plasma through blood-brain barrier disruption has assumed a more complex profile during the pathogenic cascade of MS, not only for the chemoattractant action towards neutrophils, monocytes and macrophages in the inflammatory phase [39], but also as an early trigger of microglial activation that leads to the axonal damage. Fbg appeared to be a determinant factor in the release of oxygen reactive species (ROS) in microglial cells *via* the protein interaction with the CD11b/CD18 integrin receptor, a crucial step in the mechanism of axonal damage that may be inhibited by anticoagulant drugs [40].

Fibrin deposits were also described in chronic progressive MS plaques [41], whereas a MALDI-TOF spectrometry analysis revealed the downregulation of its signal in PPMS subjects [42].

With caution due to the low number of subjects (and the significance of the p-values, mostly close to 0.05), we hypothesized that the upregulation of Fbg and FPA observed in our PrMS sample might be considered a marker of the progression onset. In support of this hypothesis, the intensities of both proteins significantly correlated with the baseline disability score. One possible explanation might be that these proteins gradually increase during the MS course, and when their levels reach a given threshold, they accumulate in a “toxic” concentration for microglia and oligodendrocytes, thus reinforcing the axonal damage. However, further studies need to be performed in order to confirm this hypothesis.

Few notes on Tβ4, a protein highly expressed in oligodendrocytes and correlated with cellular growth and regeneration mainly by modulating the availability of cellular actin monomers [43], [44]. Its function remains controversial; Morris et al [45] demonstrated that the administration of Tβ4 ameliorated EAE by exerting anti-inflammatory properties, and clinical improvements were also observed after the same treatment in case of stroke and brain injury, in support of its role in cellular plasticity. Conversely, silencing the Tβ4 gene reduced the invasiveness of the tumor cells in glioblastoma [46].

In our study, the peak intensity of Tβ4 was significantly higher and discriminated between CIS and RRMS patients and these data validated the anti-inflammatory and regenerative roles of this protein in the very early phase of the disease. However, a more focused study in a larger dataset needs to be performed in order to explain the distinctive higher level of this protein in dementia and other degenerative diseases, as was previously demonstrated by some authors from this research group [9] and confirmed in this investigation.

As often stated in the report, this study is limited by the restricted sample size especially of the PrMS subgroup, which is evident considering that some suggestive results were derived from these patients. However, we note the limited availability of samples belonging to SPMS subjects since, in clinical practice, it is more frequent that these patients are submitted to lumbar puncture before the onset of the progression.

A final note about the choice of CSF samples belonging to dementia and CIDP subjects as control group; by following international guidelines [17], this decision was made in order to verify the pathological specificity of the significant protein signals emerged from the analysis, as additional aim of the study. In our opinion, this was also essential given the relatively low numbers of investigations in the chosen proteome range <15000 Da.

Unfortunately the age range of these two groups (available in the tissue bank of the Neuroscience Department of the University of Bari) was older than the MS population. However we believe that this detail did not affect significantly the results of our investigation, since it was not built for this purpose, leaving the possible pathogenic explanation to further – less explorative, more confirmatory – studies (see below).

Above all, in our opinion this MALDI-TOF mass spectrometry evaluation has proven that this technique can generate important information for understanding the pathogenesis of a complex disease like MS, and with a relatively small sample size. The main results showed that, although not specific, the de-escalation of some Granins related to regeneration (and possibly of Tβ4) together with the accumulation of Fibrinogen and its peptides, more consistently responsible for the axonal damage, might play a distinctive role in MS progression. Validation of this preliminary hypothesis will be performed on independent MS populations and different control groups, also supported by other concomitant strategies, e.g. a large-scale dosage of the proteins of interest, in order to better understand the role of these biomarkers in the disease evolution.

## Supporting Information

Appendix S1 **Figure S1, Tables S1–S3.** Figure S1: Examples of spectra in CIS subjects with the unidentified peak signal at 3817.45 m/z. Table S1: peak signals that discriminated between the MS subgroups. Table S2: list of already identified proteins enclosed in the *m/z* range of interest in our study. Table S3: comparisons of clinical and demographic features between CIS subjects who did not converted (CIS-CIS) and those who shifted to CDMS.
(DOCX)

## References

[1] Comi G, Martinelli V, Rodegher M, Moiola L, Leocani L, et al (2013) Effects of early treatment with glatiramer acetate in patients with clinically isolated syndrome. Mult Scler 19: 1074–1083. 23234810 10.1177/1352458512469695

[2] Nagtegaal GJ, Pohl C, Wattjes MP, Hulst HE, Freedman MS, et al (2014) Interferon beta-1b reduces black holes in a randomised trial of clinically isolated syndrome. Mult Scler 20: 234–242. 23842212 10.1177/1352458513494491

[3] Trojano M, Paolicelli D, Tortorella C, Iaffaldano P, Lucchese G, et al (2011) Natural history of multiple sclerosis: have available therapies impacted long-term prognosis? Neurol Clin 29: 309–321. 21439443 10.1016/j.ncl.2010.12.008

[4] Scalfari A, Neuhaus A, Daumer M, Muraro PA, Ebers GC (2014) Onset of secondary progressive phase and long-term evolution of multiple sclerosis. J Neurol, Neurosurg Psychiatry 85: 67–75. 23486991 10.1136/jnnp-2012-304333

[5] Zastepa E, Fitz-Gerald L, Hallett M, Antel J, Bar-Or A, et al (2014) Naive CD4 T-cell activation identifies MS patients having rapid transition to progressive MS. Neurology 82: 681–690. 24453076 10.1212/WNL.0000000000000146 PMC3945666

[6] Schutzer SE, Angel TE, Liu T, Schepmoes AA, Xie F, et al (2013) Gray matter is targeted in first-attack multiple sclerosis. PloS One 8: e66117. 24039694 10.1371/journal.pone.0066117 PMC3769274

[7] Komori M, Matsuyama Y, Nirasawa T, Thiele H, Becker M, et al (2012) Proteomic pattern analysis discriminates among multiple sclerosis-related disorders. Ann Neurol 71: 614–623. 22522477 10.1002/ana.22633

[8] Dhaunchak AS, Becker C, Schulman H, De Faria O Jr, Rajasekharan S, et al (2012) Implication of perturbed axoglial apparatus in early pediatric multiple sclerosis. Ann Neurol 71: 601–613. 22473675 10.1002/ana.22693

[9] Le Pera M, Urso E, Sprovieri T, Bossio S, Aguglia U, et al (2012) Contribution of cerebrospinal fluid thymosin beta4 levels to the clinical differentiation of Creutzfeldt-Jakob disease. Arch Neurol 69: 868–872. 22431836 10.1001/archneurol.2011.3558

[10] Lublin FD, Reingold SC (1996) Defining the clinical course of multiple sclerosis: results of an international survey. National Multiple Sclerosis Society (USA) Advisory Committee on Clinical Trials of New Agents in Multiple Sclerosis. Neurology 46: 907–11. 8780061 10.1212/wnl.46.4.907

[11] Polman CH, Reingold SC, Edan G, Filippi M, Hartung HP, et al (2005) Diagnostic criteria for multiple sclerosis: 2005 revisions to the “McDonald Criteria”. Ann Neurol 58: 840–846. 16283615 10.1002/ana.20703

[12] Polman CH, Reingold SC, Banwell B, Clanet M, Cohen JA, et al (2011) Diagnostic criteria for multiple sclerosis: 2010 revisions to the McDonald criteria. Ann Neurol 69: 292–302. 21387374 10.1002/ana.22366 PMC3084507

[13] McDonald WI, Compston A, Edan G, Goodkin D, Hartung HP, et al (2001) Recommended diagnostic criteria for multiple sclerosis: guidelines from the International Panel on the diagnosis of multiple sclerosis. Ann Neurol 50(1): 121–127. 11456302 10.1002/ana.1032

[14] Teunissen CE, Petzold A, Bennett JL, Berven FS, Brundin L, et al (2009) A consensus protocol for the standardization of cerebrospinal fluid collection and biobanking. Neurology 73: 1914–1922. 19949037 10.1212/WNL.0b013e3181c47cc2 PMC2839806

[15] Poser CM, Paty DW, Scheinberg L, McDonald WI, Davis FA, et al (1983) New diagnostic criteria for multiple sclerosis: guidelines for research protocols. Ann Neurol 13: 227–231. 6847134 10.1002/ana.410130302

[16] Tortorella C, Ruggieri M, Di Monte E, Ceci E, Iaffaldano P, et al (2011) Serum and CSF N-acetyl aspartate levels differ in multiple sclerosis and neuromyelitis optica. J Neurol, Neurosurg Psychiatry 82: 1355–1359. 21622936 10.1136/jnnp.2011.241836

[17] Teunissen C, Menge T, Altintas A, Alvarez-Cermeno JC, Bertolotto A, et al (2013) Consensus definitions and application guidelines for control groups in cerebrospinal fluid biomarker studies in multiple sclerosis. Mul Scler 19(13): 1802–1809. 10.1177/1352458513488232 23695446

[18] Urso E, Le Pera M, Bossio S, Sprovieri T, Qualtieri A (2010) Quantification of thymosin beta(4) in human cerebrospinal fluid using matrix-assisted laser desorption/ionization time-of-flight mass spectrometry. Anal Biochem 402: 13–19. 20346905 10.1016/j.ab.2010.03.029

[19] Qualtieri A, Urso E, Le Pera M, Sprovieri T, Bossio S, et al (2010) Proteomic profiling of cerebrospinal fluid in Creutzfeldt-Jakob disease. Exp Rev Proteomics 7: 907–917. 10.1586/epr.10.80 21142891

[20] Anderson DR, Burnham KP, Gould WR, Cherry S (2001) Concerns about finding effects that are actually spurious. In: Biometrics, Wildlife Society Bullettin 29(1): 311–316.

[21] Bender R, Lange S (2001) Adjusting for multiple testing – when and how? J Clin Epidemiol 54: 343–349. 11297884 10.1016/s0895-4356(00)00314-0

[22] Benjamini Y, Hochberg Y (1995) Controlling the false discovery rate: A practical and powerful approach to multiple testing. Journal of the Royal Statistical Society Series B (Methodological) 57: 289–300.

[23] Lassmann H, Bruck W, Lucchinetti C (2001) Heterogeneity of multiple sclerosis pathogenesis: implications for diagnosis and therapy. Trends Mol Med 7: 115–121. 11286782 10.1016/s1471-4914(00)01909-2

[24] Filippi M, Rocca MA, Barkhof F, Bruck W, Chen JT, et al (2012) Association between pathological and MRI findings in multiple sclerosis. Lancet Neurol 11: 349–360. 22441196 10.1016/S1474-4422(12)70003-0

[25] Kappos L, Achtnichts L, Dahlke F, Kuhle J, Naegelin Y, et al (2005) Genomics and proteomics: role in the management of multiple sclerosis. J Neurol 252 Suppl 3: iii21–7. 16170496 10.1007/s00415-005-2013-3

[26] International Multiple Sclerosis Genetics C, Wellcome Trust Case Control C, Sawcer S, Hellenthal G, Pirinen M, Spencer CC, Patsopoulos NA, et al (2011) Genetic risk and a primary role for cell-mediated immune mechanisms in multiple sclerosis. Nature 476: 214–219. 21833088 10.1038/nature10251 PMC3182531

[27] Patsopoulos NA, Bayer Pharma MSGWG, Steering Committees of Studies Evaluating I-b, ANZgene Consortium, GeneMSA, et al (2011) Genome-wide meta-analysis identifies novel multiple sclerosis susceptibility loci. Ann Neurol 70: 897–912. 22190364 10.1002/ana.22609 PMC3247076

[28] Disanto G, Handel AE, Damoiseaux J, Hupperts R, Giovannoni G, et al (2013) Vitamin D supplementation and antibodies against the Epstein-Barr virus in multiple sclerosis patients. Mult Scler 19: 1679–1680. 23828870 10.1177/1352458513494494

[29] Verheul F, Smolders J, Trojano M, Lepore V, Zwanikken C, et al (2013) Fluctuations of MS births and UV-light exposure. Acta Neurol Scand 127: 301–8. 22970985 10.1111/ane.12007

[30] Dagley LF, Emili A, Purcell AW (2013) Application of quantitative proteomics technologies to the biomarker discovery pipeline for multiple sclerosis. Proteomics Clin Appl 7: 91–108. 23112123 10.1002/prca.201200104

[31] Stoop MP, Singh V, Dekker LJ, Titulaer MK, Stingl C, et al (2010) Proteomics comparison of cerebrospinal fluid of relapsing remitting and primary progressive multiple sclerosis. PloS One 5: e12442. 20805994 10.1371/journal.pone.0012442 PMC2929207

[32] Comabella M, Fernandez M, Martin R, Rivera-Vallve S, Borras E, et al (2010) Cerebrospinal fluid chitinase 3-like 1 levels are associated with conversion to multiple sclerosis. Brain 133: 1082–1093. 20237129 10.1093/brain/awq035

[33] Fischer-Colbrie R, Kirchmair R, Kahler CM, Wiedermann CJ, Saria A (2005) Secretoneurin: a new player in angiogenesis and chemotaxis linking nerves, blood vessels and the immune system. Curr Protein Pept Sci 6: 373–85. 16101435 10.2174/1389203054546334

[34] Mattsson N, Ruetschi U, Podust VN, Stridsberg M, Li S, et al (2007) Cerebrospinal fluid concentrations of peptides derived from chromogranin B and secretogranin II are decreased in multiple sclerosis. J Neurochem 103: 1932–1939. 17953655 10.1111/j.1471-4159.2007.04985.x

[35] Mattsson N, Johansson P, Hansson O, Wallin A, Johansson JO, et al (2010) Converging pathways of chromogranin and amyloid metabolism in the brain. J Alzheimers Dis 20: 1039–1049. 20413871 10.3233/JAD-2010-091651

[36] Helwig M, Hoshino A, Berridge C, Lee SN, Lorenzen N, et al (2013) The neuroendocrine protein 7B2 suppresses the aggregation of neurodegenerative disease-related proteins. J Biol Chemistry 288: 1114–1124. 10.1074/jbc.M112.417071 PMC3542996 23172224

[37] Valis M, Talab R, Stourac P, Andrys C, Masopust J (2008) Tau protein, phosphorylated tau protein and beta-amyloid42 in the cerebrospinal fluid of multiple sclerosis patients. Neuro Endocrinol Lett 29: 971–6. 19112391

[38] Mai W, Hu X, Lu Z, Peng F, Wang Y (2011) Cerebrospinal fluid levels of soluble amyloid precursor protein and beta-amyloid 42 in patients with multiple sclerosis, neuromyelitis optica and clinically isolated syndrome. J Int Med Res 39: 2402–2413. 22289560 10.1177/147323001103900641

[39] Jennewein C, Tran N, Paulus P, Ellinghaus P, Eble JA, et al (2011) Novel aspects of fibrin(ogen) fragments during inflammation. Mol Med 17: 568–573. 21210072 10.2119/molmed.2010.00146 PMC3105136

[40] Davalos D, Ryu JK, Merlini M, Baeten KM, Le Moan N, et al (2012) Fibrinogen-induced perivascular microglial clustering is required for the development of axonal damage in neuroinflammation. Nature Commun 3: 1227. 23187627 10.1038/ncomms2230 PMC3514498

[41] Gveric D, Hanemaaijer R, Newcombe J, van Lent NA, Sier CF, et al (2001) Plasminogen activators in multiple sclerosis lesions: implications for the inflammatory response and axonal damage. Brain 124: 1978–1988. 11571216 10.1093/brain/124.10.1978

[42] Teunissen CE, Koel-Simmelink MJ, Pham TV, Knol JC, Khalil M, et al (2011) Identification of biomarkers for diagnosis and progression of MS by MALDI-TOF mass spectrometry. Mult Scler 17: 838–850. 21505015 10.1177/1352458511399614

[43] Zhang J, Zhang ZG, Morris D, Li Y, Roberts C, et al (2009) Neurological functional recovery after thymosin beta4 treatment in mice with experimental auto encephalomyelitis. Neuroscience 164: 1887–1893. 19782721 10.1016/j.neuroscience.2009.09.054 PMC2784109

[44] Mollinari C, Ricci-Vitiani L, Pieri M, Lucantoni C, Rinaldi AM, et al (2009) Downregulation of thymosin beta4 in neural progenitor grafts promotes spinal cord regeneration. J Cell Sci 122: 4195–4207. 19861493 10.1242/jcs.056895

[45] Morris DC, Zhang ZG, Zhang J, Xiong Y, Zhang L, et al (2012) Treatment of neurological injury with thymosin beta4. Ann N Y Acad Sci 1269: 110–116. 23045978 10.1111/j.1749-6632.2012.06651.x PMC3471669

[46] Wirsching HG, Krishnan S, Florea AM, Frei K, Krayenbuhl N, et al (2014) Thymosin beta 4 gene silencing decreases stemness and invasiveness in glioblastoma. Brain 137: 433–448. 24355709 10.1093/brain/awt333

