# Supplementary material for: Proteomic Profiling in Multiple Sclerosis Clinical Courses Reveals Potential Biomarkers of Neurodegeneration
Source: PLoS One. 2014 Aug 6;9(8):e103984. doi: 10.1371/journal.pone.0103984 (PMC4123901; doi:10.1371/journal.pone.0103984)
Supplement: Appendix S1 — Figure S1, Tables S1–S3. Figure S1: Examples of spectra in CIS subjects with the unidentified peak signal at 3817.45 m/z. Table S1: peak signals that discriminated between the MS subgroups. Table S2: list of already identified proteins enclosed in the m/z range of interest in our study. Table S3: comparisons of clinical and demographic features between CIS subjects who did not converted (CIS-CIS) and those who shifted to CDMS. (DOCX) [file pone.0103984.s001.docx]

**Figure S1:** Examples of spectra in CIS subjects with the unidentified peak signal at 3817.45 m/z, absent in A but showing high intensity (19% of total peak area) in B.*
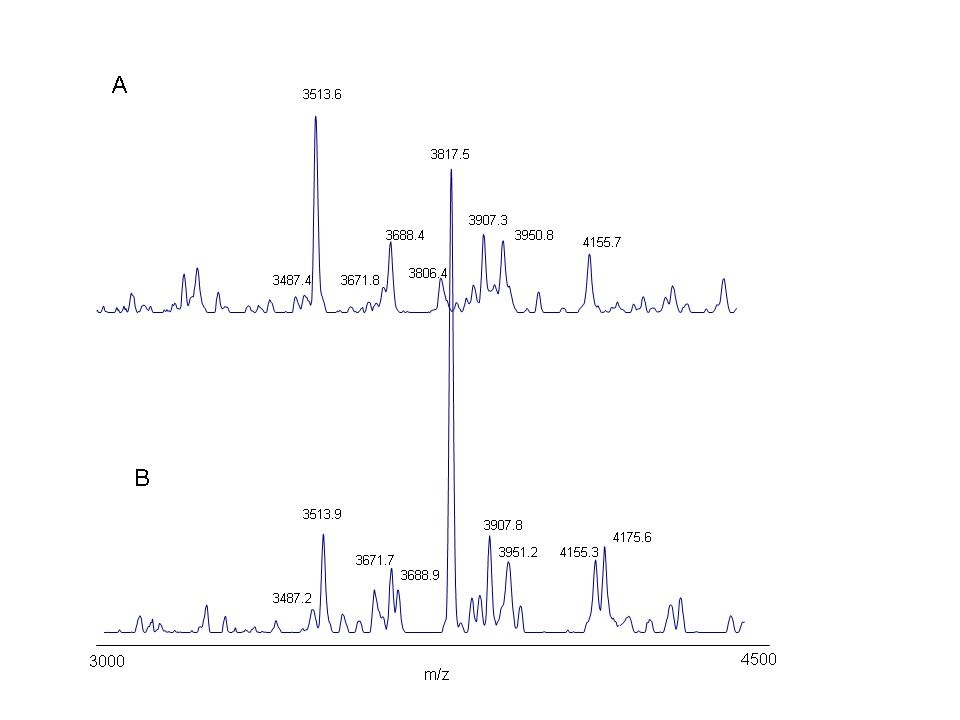
*

**Table S1**: peak signals that discriminated between the MS subgroups

***A. CIS versus RRMS***

| ***m/z (Average)*** | **CIS** | **Mean** | **SD** | **RRMS** | **Mean** | **SD** |
| --- | --- | --- | --- | --- | --- | --- |
| 1248.074939 | 24 | ,0043956170 | ,01608135939 | 16 | ,2730230309 | ,69791957685 |
| 1288.870701 | 24 | ,0076831817 | ,02372535237 | 16 | ,4491418711 | 1,16061094432 |
| 1334.755157 | 24 | ,0369850694 | ,04736029742 | 16 | ,2220258534 | ,49840528962 |
| 1526.623702 | 24 | ,0211785185 | ,05709385501 | 16 | ,0878445221 | ,11619563093 |
| 1834.40706 | 24 | ,0152025037 | ,03669944317 | 16 | ,0513241631 | ,06967681823 |
| 2701.92928 | 24 | ,0044566857 | ,02183321186 | 16 | ,0591029347 | ,11690034086 |
| 2910.336495 | 24 | ,0006406393 | ,00313847895 | 16 | ,0056246965 | ,01163998875 |
| 2915.739432 | 24 | ,0242342333 | ,08884616370 | 16 | ,0374017289 | ,08336442382 |
| 3019.764723 | 24 | ,0118082475 | ,02727455485 | 16 | ,0328281709 | ,04908247990 |
| 3474.865065 | 24 | ,0104625423 | ,01517862177 | 16 | ,0024891896 | ,00995675825 |
| 4936.619888 | 24 | ,1759002220 | ,60754748951 | 16 | ,0208093247 | ,03856337313 |
| 4964.387264 | 24 | ,3186118953 | ,27567887083 | 16 | ,1287655847 | ,15191439560 |
| 5184.333476 | 24 | ,0000000000 | ,00000000000 | 16 | ,0079667568 | ,01784002893 |
| 6819.287481 | 24 | ,5714298899 | ,36803933805 | 16 | ,3054113004 | ,30285926509 |
| 7765.369373 | 24 | ,0006076227 | ,00297673118 | 16 | ,6204875002 | 1,42819357016 |
| 8181.617374 | 24 | 2,0571147249 | ,41890462572 | 16 | 1,5146313396 | 1,25566770185 |
| 11119.03492 | 24 | ,0107517934 | ,04064579333 | 16 | 4,0248397304 | 8,12822944192 |

***B. CIS versus PrMS***

| ***m/z (Average)*** | **CIS** | **Mean** | **SD** | **PrMS** | **Mean** | **SD** |
| --- | --- | --- | --- | --- | --- | --- |
| 1201.061821 | 24 | .0302578705 | .03760880136 | 11 | .0000000000 | .00000000000 |
| 1261.131941 | 24 | .0611380525 | .06462398389 | 11 | .0124475933 | .02828583412 |
| 1284.077443 | 24 | .2214851561 | .57073189552 | 11 | .0000000000 | .00000000000 |
| 1286.738767 | 24 | .0444783325 | .10425218271 | 11 | .0000000000 | .00000000000 |
| 1295.947223 | 24 | .4558312301 | .99232238050 | 11 | .0393388994 | .07640500183 |
| 1314.331974 | 24 | .0391474006 | .05256013473 | 11 | .0048633745 | .01107316705 |
| 1334.755157 | 24 | .0369850694 | .04736029742 | 11 | .0143964912 | .04774775955 |
| 1387.617302 | 24 | .0152177338 | .04175248346 | 11 | .0000000000 | .00000000000 |
| 1545.682571 | 24 | .1873967768 | .50928665031 | 11 | 1.7757129178 | 1.66411287357 |
| 1582.822918 | 24 | .0122402865 | .02398026812 | 11 | .0529516310 | .05817684173 |
| 1694.407821 | 24 | .1294974116 | .17440123896 | 11 | .5327895168 | .49390616939 |
| 1756.729638 | 24 | .7148920718 | .37604474619 | 11 | 1.7712313234 | 1.33503967956 |
| 1799.619328 | 24 | .0345710178 | .04785869451 | 11 | .0107676269 | .02440766782 |
| 1805.088037 | 24 | .0980303707 | .16325228524 | 11 | .7305054989 | .73449187014 |
| 2114.366848 | 24 | .2688096517 | .33645088732 | 11 | .7461977265 | .47525010689 |
| 2118.377757 | 24 | .0354653526 | .05591364771 | 11 | .0046988835 | .01045568977 |
| 2141.90573 | 24 | .1339553110 | .16814049620 | 11 | .4009867299 | .42487156791 |
| 2245.29418 | 24 | .5213764365 | .38768545053 | 11 | 1.1420448304 | .71718715385 |
| 2390.95868 | 24 | .3382345549 | .33583849216 | 11 | .6675943566 | .47297359251 |
| 2508.418159 | 24 | .2255466072 | .55382502410 | 11 | .3950804556 | .34814680062 |
| 2765.000697 | 24 | .3430825313 | .29902303968 | 11 | .1220251352 | .17132423946 |
| 2768.781538 | 24 | .0475045719 | .09531967007 | 11 | .4402677405 | .60479789378 |
| 2861.559061 | 24 | .0678318513 | .09173265722 | 11 | .5236633648 | .60277074401 |
| 2904.143722 | 24 | .0219134902 | .03570266479 | 11 | .1480526734 | .17380329250 |
| 2950.477469 | 24 | .0414925658 | .06064089121 | 11 | .1382813387 | .15054702180 |
| 4154.534971 | 24 | 2.7538421405 | .87807262746 | 11 | 1.6253433826 | .70940001020 |
| 4351.397657 | 24 | 1.2804451983 | .57571014068 | 11 | .6714086058 | .52805989732 |
| 4808.764943 | 24 | .9891419233 | .55817615090 | 11 | .4077260370 | .27117478694 |
| 4951.225718 | 24 | .0260376620 | .03514586295 | 11 | .0043494095 | .01442535922 |
| 5455.468774 | 24 | .1299757454 | .19163176438 | 11 | .0029291472 | .00971488216 |
| 5570.996112 | 24 | .0603801188 | .08025275474 | 11 | .0180446542 | .04759792371 |
| 6819.287481 | 24 | .5714298899 | .36803933805 | 11 | .1654952461 | .12652750220 |
| 6972.378809 | 24 | 3.0059243984 | 2.06519448825 | 11 | 1.4835082028 | 1.32180223475 |
| 8181.617374 | 24 | 2.0571147249 | .41890462572 | 11 | 1.2632765902 | .50251112949 |
| 8293.650355 | 24 | .5370349115 | .19122100997 | 11 | .3884480093 | .14597885078 |

***C. CIS versus dementias***

| ***m/z (Average)*** | **CIS** | **Mean** | **SD** | **dementias** | **Mean** | **SD** |
| --- | --- | --- | --- | --- | --- | --- |
| 1207.01173 | 24 | ,0227302380 | ,03351910250 | 7 | ,0707176867 | ,02773083002 |
| 1261.131941 | 24 | ,0611380525 | ,06462398389 | 7 | ,0093201666 | ,01779748061 |
| 1609.99534 | 24 | ,1104422105 | ,19584711569 | 7 | ,0059416213 | ,01572005231 |
| 1827.128758 | 24 | ,2510399157 | ,26823866299 | 7 | ,0198852870 | ,03685357248 |
| 1866.130189 | 24 | ,0126066720 | ,03766369119 | 7 | ,1533038530 | ,24176673525 |
| 1940.503656 | 24 | ,0751348871 | ,11985251475 | 7 | ,0000000000 | ,00000000000 |
| 2082.822983 | 24 | ,0339952978 | ,06630462943 | 7 | ,2073200120 | ,14512248816 |
| 2096.950639 | 24 | ,0182335663 | ,03784749621 | 7 | ,0000000000 | ,00000000000 |
| 2114.366848 | 24 | ,2688096517 | ,33645088732 | 7 | ,0779108886 | ,17405543627 |
| 2118.377757 | 24 | ,0354653526 | ,05591364771 | 7 | ,1784171460 | ,17585244350 |
| 2245.29418 | 24 | ,5213764365 | ,38768545053 | 7 | ,1521456654 | ,21494227284 |
| 2280.231937 | 24 | ,0290089274 | ,07864735719 | 7 | ,0444394594 | ,03613436058 |
| 2316.077043 | 24 | ,7732701883 | ,58646186636 | 7 | ,2615225281 | ,27405619956 |
| 2329.128792 | 24 | ,0318647277 | ,10759367080 | 7 | ,0954701943 | ,09339711283 |
| 2378.589137 | 24 | ,2444937421 | ,23758076365 | 7 | ,5659263180 | ,34656525834 |
| 2390.95868 | 24 | ,3382345549 | ,33583849216 | 7 | ,0550897299 | ,06841349453 |
| 2412.608445 | 24 | ,1203406423 | ,16196970745 | 7 | ,0000000000 | ,00000000000 |
| 2448.567903 | 24 | ,3272009584 | ,27976726915 | 7 | ,5644311167 | ,11462546606 |
| 2604.408315 | 24 | ,2279831120 | ,22135946373 | 7 | ,0014464207 | ,00382686950 |
| 2681.931754 | 24 | ,1543311858 | ,27787981096 | 7 | ,0000000000 | ,00000000000 |
| 2793.543547 | 24 | ,2262703921 | ,18974629121 | 7 | ,0668983994 | ,06535619259 |
| 3204.091883 | 24 | ,4521777256 | ,44947586593 | 7 | ,8597843941 | ,39749498493 |
| 3235.048571 | 24 | ,4680979328 | ,65627826483 | 7 | 1,0961231160 | ,64429298598 |
| 3354.341171 | 24 | ,3843916735 | ,28182204198 | 7 | ,0856850671 | ,13298203219 |
| 3669.917046 | 24 | ,5537699727 | ,85251366422 | 7 | ,0000000000 | ,00000000000 |
| 3676.711569 | 24 | ,0682198219 | ,26675079551 | 7 | ,7165629754 | ,43101415806 |
| 3688.85139 | 24 | 2,6820345844 | 1,06347221690 | 7 | 1,7210849620 | ,83539900705 |
| 3817.361 | 24 | 1,9467377913 | 3,90958097942 | 7 | ,1525685420 | ,35283158456 |
| 3883.341415 | 24 | 1,0295904242 | ,53067577994 | 7 | ,5399586326 | ,13181864936 |
| 3952.462769 | 24 | 4,7178539525 | 1,78669344807 | 7 | 3,1615286017 | 1,29535663938 |
| 4091.331943 | 24 | ,2262529465 | ,18373661329 | 7 | ,4751580746 | ,12904429552 |
| 4154.534971 | 24 | 2,7538421405 | ,87807262746 | 7 | 1,7869526136 | ,87005655406 |
| 4281.2447 | 24 | ,4268453620 | ,31228474549 | 7 | ,8004916556 | ,14336031936 |
| 4351.397657 | 24 | 1,2804451983 | ,57571014068 | 7 | ,5073817630 | ,25763209828 |
| 4683.557943 | 24 | ,0418250347 | ,08377285912 | 7 | ,0760391990 | ,04322345609 |
| 4808.764943 | 24 | ,9891419233 | ,55817615090 | 7 | ,2565533813 | ,18430463816 |
| 5047.122095 | 24 | ,1635690932 | ,13091043568 | 7 | ,3993520526 | ,12643210364 |
| 5064.306122 | 24 | ,8287013236 | ,47056721388 | 7 | 1,2396583786 | ,18977354963 |
| 5084.382633 | 24 | ,0819831159 | ,15775400179 | 7 | ,0000000000 | ,00000000000 |
| 5258.836336 | 24 | ,1124609418 | ,14368765761 | 7 | ,4047713909 | ,07796996603 |
| 5570.996112 | 24 | ,0603801188 | ,08025275474 | 7 | ,1359370167 | ,11639689974 |
| 6819.287481 | 24 | ,5714298899 | ,36803933805 | 7 | ,1219574967 | ,08581306831 |
| 8564.962881 | 24 | ,4397356419 | ,17028324308 | 7 | ,8045240253 | ,14996186139 |
| 8603.21 | 24 | ,1392803792 | ,23276921921 | 7 | 1,2293998123 | 1,29680588985 |

***D. CIS versus CIDP***

| ***m/z (Average)*** | **CIS** | **Mean** | **SD** | **CIDP** | **Mean** | **SD** |
| --- | --- | --- | --- | --- | --- | --- |
| 1207.01173 | 24 | ,0227302380 | ,03351910250 | 11 | ,2235972670 | ,31539566819 |
| 1261.131941 | 24 | ,0611380525 | ,06462398389 | 11 | ,0156886596 | ,03036936769 |
| 1308.973467 | 24 | ,3303539088 | ,45607287328 | 11 | ,8859857597 | ,42928136631 |
| 1424.537736 | 24 | ,0892001865 | ,08473465012 | 11 | ,0247924244 | ,04404398860 |
| 1448.023939 | 24 | ,0247463659 | ,05475940879 | 11 | ,0793571025 | ,07571462323 |
| 1450.68053 | 24 | ,0213330169 | ,04925685567 | 11 | ,1360888650 | ,24958817312 |
| 1466.39831 | 24 | ,3753978389 | ,59484135404 | 11 | 1,8413219527 | 1,99253493367 |
| 1512.344439 | 24 | ,0494219909 | ,06146271126 | 11 | ,0119340344 | ,02086346665 |
| 1615.979821 | 24 | ,1075754725 | ,21202140606 | 11 | ,2842729458 | ,28133032485 |
| 1697.189965 | 24 | ,0375661623 | ,07897898209 | 11 | ,0000000000 | ,00000000000 |
| 1736.327273 | 24 | ,2374665319 | ,38290509415 | 11 | ,0216616456 | ,03738214445 |
| 1763.867148 | 24 | ,2071135338 | ,29775559835 | 11 | ,5953216849 | ,65602596139 |
| 1827.128758 | 24 | ,2510399157 | ,26823866299 | 11 | ,0712543420 | ,10459656770 |
| 1866.130189 | 24 | ,0126066720 | ,03766369119 | 11 | ,9417953362 | 2,13861911794 |
| 1940.503656 | 24 | ,0751348871 | ,11985251475 | 11 | ,0102613707 | ,02732818844 |
| 1982.959266 | 24 | ,0424847852 | ,07049440798 | 11 | ,0057109055 | ,01894093061 |
| 2009.63628 | 24 | ,0410040660 | ,07567506463 | 11 | ,0000000000 | ,00000000000 |
| 2073.724431 | 24 | ,0907436563 | ,12884153392 | 11 | ,0392247371 | ,10521384547 |
| 2082.822983 | 24 | ,0339952978 | ,06630462943 | 11 | ,5112689867 | ,57616504243 |
| 2141.90573 | 24 | ,1339553110 | ,16814049620 | 11 | ,0297205448 | ,04634615435 |
| 2227.40837 | 24 | ,1559474623 | ,34465121927 | 11 | 1,3098414113 | 1,58580227223 |
| 2277.741991 | 24 | ,0060746000 | ,02066881469 | 11 | ,1043806223 | ,18512132764 |
| 2412.608445 | 24 | ,1203406423 | ,16196970745 | 11 | ,0153990991 | ,02118379223 |
| 2486.847485 | 24 | ,1813094041 | ,22889187328 | 11 | ,0344758902 | ,06265630557 |
| 2524.121457 | 24 | 1,4736624662 | ,60382291565 | 11 | ,9415112995 | ,39288256797 |
| 2660.556718 | 24 | ,0014194946 | ,00695407484 | 11 | ,0541265167 | ,07741994254 |
| 2754.6938 | 24 | ,2234296295 | ,24315915636 | 11 | ,6738631392 | ,54966105515 |
| 2768.781538 | 24 | ,0475045719 | ,09531967007 | 11 | ,5107138325 | ,71652573688 |
| 2931.630094 | 24 | ,0515935901 | ,16107389524 | 11 | ,1043038459 | ,14964925042 |
| 3047.276928 | 24 | ,0221124855 | ,05135643041 | 11 | ,0000000000 | ,00000000000 |
| 3284.158649 | 24 | ,5031335173 | ,33765065630 | 11 | ,1507733240 | ,13876279062 |
| 3669.917046 | 24 | ,5537699727 | ,85251366422 | 11 | ,0000000000 | ,00000000000 |
| 3676.711569 | 24 | ,0682198219 | ,26675079551 | 11 | ,9934354624 | 1,13710139834 |
| 3952.462769 | 24 | 4,7178539525 | 1,78669344807 | 11 | 2,7296436929 | ,99739613388 |
| 4134.434496 | 24 | ,1408238777 | ,12750207081 | 11 | ,3775837452 | ,23503072871 |
| 4154.534971 | 24 | 2,7538421405 | ,87807262746 | 11 | 1,4089988915 | ,51668610696 |
| 4281.2447 | 24 | ,4268453620 | ,31228474549 | 11 | ,8904138843 | ,58116601556 |
| 4304.109773 | 24 | ,0279057110 | ,04978084220 | 11 | ,3789739356 | ,41724531766 |
| 4332.023469 | 24 | ,5627752750 | ,51224920371 | 11 | 1,5131122864 | ,84726182086 |
| 4351.397657 | 24 | 1,2804451983 | ,57571014068 | 11 | ,6106435667 | ,52045442722 |
| 4587.095706 | 24 | 1,4684654207 | ,80787162404 | 11 | ,7680762536 | ,50585735524 |
| 4605.886308 | 24 | ,4617443918 | ,35726017551 | 11 | ,2030155877 | ,34813781122 |
| 4640.960572 | 24 | ,2233291598 | ,22885128582 | 11 | ,0691499115 | ,11920511011 |
| 4705.67954 | 24 | ,0033206753 | ,01626792034 | 11 | ,0379006589 | ,08817153350 |
| 4740.05293 | 24 | ,1444422060 | ,22473836218 | 11 | ,5993362068 | ,79839928671 |
| 4808.764943 | 24 | ,9891419233 | ,55817615090 | 11 | ,2347747354 | ,17954955860 |
| 4951.225718 | 24 | ,0260376620 | ,03514586295 | 11 | ,0063614489 | ,02109853915 |
| 5190.933915 | 24 | ,0245346380 | ,05054456582 | 11 | ,0000000000 | ,00000000000 |
| 5570.996112 | 24 | ,0603801188 | ,08025275474 | 11 | ,0126547232 | ,04197096862 |
| 6819.287481 | 24 | ,5714298899 | ,36803933805 | 11 | ,1908067066 | ,16101666020 |
| 8181.617374 | 24 | 2,0571147249 | ,41890462572 | 11 | ,5921670471 | ,78892321722 |
| 8293.650355 | 24 | ,5370349115 | ,19122100997 | 11 | ,3128896712 | ,22918247473 |

***E. MS versus OND***

| ***m/z (Average)*** | **MS** | **Mean** | **SD** | **OND** | **Mean** | **SD** |
| --- | --- | --- | --- | --- | --- | --- |
| 1207.01173 | 51 | .0453785392 | .11391509597 | 18 | .1641440969 | .25429709366 |
| 1261.131941 | 51 | .1554656805 | .70761913010 | 18 | .0132120234 | .02577844936 |
| 1308.973467 | 51 | .3284400186 | .43330917996 | 18 | .6896354042 | .44448881651 |
| 1337.561443 | 51 | .2313167981 | .37500507684 | 18 | .1085162093 | .32853927667 |
| 1448.023939 | 51 | .0259809105 | .04896502413 | 18 | .0582971819 | .06575555726 |
| 1458.610535 | 51 | .0191967828 | .03452620551 | 18 | .0023798061 | .01009666200 |
| 1512.344439 | 51 | .1175535383 | .41366623317 | 18 | .0157830643 | .02376867570 |
| 1553.430676 | 51 | .0500957110 | .09077955160 | 18 | .0081073036 | .03003980716 |
| 1582.822918 | 51 | .0199437981 | .03648523268 | 18 | .0066257871 | .02202141944 |
| 1615.979821 | 51 | .1419275037 | .33604354726 | 18 | .2256555733 | .25637833155 |
| 1694.407821 | 51 | .2438766449 | .32639724902 | 18 | .0877428236 | .23556188439 |
| 1697.189965 | 51 | .0327819813 | .07418860543 | 18 | .0016251288 | .00689483771 |
| 1699.703685 | 51 | .0222243426 | .05391169124 | 18 | .0000000000 | .00000000000 |
| 1736.327273 | 51 | .2615674266 | .39318198761 | 18 | .0419943157 | .07074456581 |
| 1823.694325 | 51 | .0557084383 | .13805548047 | 18 | .0045542447 | .01325703527 |
| 1827.128758 | 51 | .2832567177 | .39347131678 | 18 | .0512774873 | .08705699560 |
| 1866.130189 | 51 | .0594128372 | .34041952145 | 18 | .6351597594 | 1.69336419748 |
| 1940.503656 | 51 | .0817518560 | .12025890237 | 18 | .0062708377 | .02158257817 |
| 1957.48634 | 51 | .1778103107 | .21549189897 | 18 | .0479268953 | .08358367174 |
| 1977.731404 | 51 | .2619463858 | .19652603176 | 18 | .1510103873 | .14820782281 |
| 2009.63628 | 51 | .0418705643 | .10592437653 | 18 | .0000000000 | .00000000000 |
| 2082.822983 | 51 | .0442610429 | .07130815299 | 18 | .3930666077 | .47534675837 |
| 2114.366848 | 51 | .3419769423 | .40147220653 | 18 | .1473737533 | .19678654740 |
| 2118.377757 | 51 | .0522284363 | .14084832572 | 18 | .1430528182 | .17247579205 |
| 2141.90573 | 51 | .1988516030 | .26132665712 | 18 | .0337458078 | .04430803623 |
| 2212.67032 | 51 | .0307703487 | .08506522923 | 18 | .1621839515 | .33805111377 |
| 2329.128792 | 51 | .0239013584 | .07730533443 | 18 | .0775822403 | .11947112572 |
| 2390.95868 | 51 | .3619390709 | .37767068199 | 18 | .1416817047 | .13580582994 |
| 2412.608445 | 51 | .1209119672 | .17865841275 | 18 | .0094105606 | .01799007834 |
| 2448.567903 | 51 | .3064763283 | .26519470447 | 18 | .4783596300 | .21018377013 |
| 2476.253541 | 51 | .4679814098 | .39361409364 | 18 | 1.1083312161 | .94346031403 |
| 2508.418159 | 51 | .2265270876 | .43538273256 | 18 | .0693508196 | .13532317667 |
| 2604.408315 | 51 | .2453716150 | .23439196413 | 18 | .1190737034 | .25785830263 |
| 2628.641849 | 51 | .5743974756 | .57731796881 | 18 | .8698421838 | .62055606683 |
| 2660.556718 | 51 | .0149977321 | .09613376541 | 18 | .0330773158 | .06529165077 |
| 2681.931754 | 51 | .1119183730 | .22997217181 | 18 | .0108371063 | .02818794240 |
| 2754.6938 | 51 | .2280484590 | .25650879102 | 18 | .5595295429 | .47490153801 |
| 2876.12385 | 51 | .0176467545 | .06429197288 | 18 | .0000000000 | .00000000000 |
| 3047.276928 | 51 | .0551821557 | .21412516114 | 18 | .0000000000 | .00000000000 |
| 3117.195914 | 51 | .0142756464 | .03850825304 | 18 | .0000000000 | .00000000000 |
| 3204.091883 | 51 | .3747080067 | .39610587775 | 18 | .6290854468 | .39332809395 |
| 3235.048571 | 51 | .3749994223 | .56519738288 | 18 | .7767248452 | .61669338522 |
| 3284.158649 | 51 | .4154270058 | .33701980281 | 18 | .1973248307 | .17342872616 |
| 3300.097815 | 51 | .1454034555 | .23652381306 | 18 | .0673357791 | .16745502564 |
| 3354.341171 | 51 | .4147215893 | .33942825484 | 18 | .1733070617 | .16455826397 |
| 3486.752201 | 51 | .3381620356 | .48272947768 | 18 | .7166758350 | .61126864642 |
| 3669.917046 | 51 | .4216585911 | .74557663910 | 18 | .0000000000 | .00000000000 |
| 3676.711569 | 51 | .1540475540 | .63253905270 | 18 | .8857628286 | .91948075554 |
| 3688.85139 | 51 | 2.7664924224 | 1.14858161664 | 18 | 1.9311461078 | .76332787414 |
| 3817.361 | 51 | 2.0532269632 | 3.38181153626 | 18 | .4554222589 | .57635450616 |
| 3883.341415 | 51 | .9408250496 | .58660468900 | 18 | .6457732746 | .29060574940 |
| 3952.462769 | 51 | 4.6935759849 | 1.67054355543 | 18 | 2.8975989352 | 1.10649404181 |
| 4035.764127 | 51 | .4367644960 | .32232616982 | 18 | .6587217277 | .40153593742 |
| 4091.331943 | 51 | .1943921138 | .17029562314 | 18 | .3845634643 | .20856657840 |
| 4154.534971 | 51 | 2.6283671416 | 1.28025560068 | 18 | 1.5559808946 | .67835060001 |
| 4281.2447 | 51 | .4851124503 | .82169796734 | 18 | .8554441287 | .45603422249 |
| 4304.109773 | 51 | .0318275471 | .05155680471 | 18 | .3781404715 | .41271969637 |
| 4332.023469 | 51 | .5730388291 | .52037219585 | 18 | 1.2182533063 | .76480890860 |
| 4351.397657 | 51 | 1.0769383008 | .61782046769 | 18 | .5704861986 | .43063481468 |
| 4449.632748 | 51 | .0305124631 | .13320416731 | 18 | .4191089739 | .73178177706 |
| 4587.095706 | 51 | 1.3411263409 | .78772012258 | 18 | .8505189581 | .52156913511 |
| 4605.886308 | 51 | .4950933416 | .42049654444 | 18 | .2580627311 | .35676226428 |
| 4683.557943 | 51 | .0385527700 | .07832535847 | 18 | .0861568155 | .11008330796 |
| 4705.67954 | 51 | .0051463667 | .01805989952 | 18 | .0355335864 | .07856528228 |
| 4808.764943 | 51 | .8242485791 | .61037324986 | 18 | .2432442088 | .17627161997 |
| 4936.619888 | 51 | .0957826363 | .42040720335 | 18 | .0877354718 | .09123193253 |
| 5047.122095 | 51 | .1424802736 | .13545020400 | 18 | .2956621222 | .18992815275 |
| 5084.382633 | 51 | .0659974616 | .14843314721 | 18 | .0047456339 | .01386595205 |
| 5258.836336 | 51 | .0817297918 | .12356644720 | 18 | .3160572310 | .21735781576 |
| 6819.287481 | 51 | .4004185465 | .34942076988 | 18 | .1640320139 | .13799499685 |
| 6949.436711 | 51 | .0717572176 | .44170999019 | 18 | .0000000000 | .00000000000 |
| 7658.27465 | 51 | .3516689419 | .40213297440 | 18 | .6567128622 | .53706829858 |
| 7738.287521 | 51 | .1192999713 | .13095455446 | 18 | .2705580996 | .19630697178 |
| 8181.617374 | 51 | 1.7157038691 | .84743979588 | 18 | 1.1076089704 | .92640482361 |
| 8603.21 | 51 | .1696521644 | .26008035427 | 18 | .8276914917 | 1.01646229462 |

**Table S2:** list of already identified proteins enclosed in the *m/z* range of interest in our study; in parenthesis the peptide aminoacidic position inside the native protein.

| ***m/z (Average)*** | **Protein name** | **Acc.no.** | **Reference** |
| --- | --- | --- | --- |
| 1466,39831 | Fibrinopeptide A (21-35) | P02671 | Stark M., J.Chromatography B 2001 754 357-367 |
| 1553,430676 | Fibrinopeptide B (31-44) * | P02675 | Holtta M et al., PloSOne 2012,7,e42555 |
| 1617,106423 | Fibrinopeptide A (20-35) ** | P02671 | Desiderio J., Mass Spectrometry 2005, 40, 176-181 |
| 1776,771052 | Complement 3f (1305-1319) | P01024 | Selle H et al, Comb Chem High Throughput Screen 2005 8, 801-6 |
| 2189,667907 | Testican (419-437) | Q08629 | Stark M, J.Chromatography B 2001 754 357-367 |
| 2768,781538 | Fibrinogen (576-600) | P02671 | Holtta M et al, PloSOne 2012,7,e42555 |
| 3204,091883 | Chromogranin B (273-303) | P05060 | Stark M, J.Chromatography B 2001 754 357-367 |
| 3513,260958 | Protein 7B2 (182-212) | P05408 | Stark M, J.Chromatography B 2001 754 357-367 |
| 3907,600017 | Chromogranin A (97-131) | P10645 | Mikko Holtta, PloSOne 2012,7,e42555 |
| 4154,534971 | Secretogranin II (529-566) | P13521 | Holtta M. et al, PloSOne 2012,7,e42555 |
| 4964,387264 | Thymosin ß4 | P62328 | Qualtieri et al, Proteomics Clinical Application 2009, 3, 574-583 |
| 7735,287521 | Osteopontin (249-314) ** | P10451 | Lamerz, Proteomics 2005, 5, 2789-2798 |
| 8564,962881 | Free Ubiquitin | P62988 | Qualtieri et al, Proteomics Clinical Application 2009, 3, 574-583 |
| 11730,42677 | Free  microglobulin | **P61769** | Biroccio A et al, Proteomics 2006, 6, 2305-2313 |

* Peptide Gln modified (pyroglutamic); ** Peptide phosphorylated.

**Table S3:** comparisons of clinical and demographic features between CIS subjects who did not converted at the end of follow up (CIS-CIS) and those who shifted to CDMS, and peak signals that resulted significantly differed between the two subgroups (*identification in progress*).

|  | **CIS-CIS (n. 5)** | **CDMS (n. 19)** | **p-value** | **FDR** |
| --- | --- | --- | --- | --- |
|  |  |  |  |  |
| female/male | 3/2 | 15/4 | 0.3 | -- |
| mean age at onset (SD) years | 30.3 (6.9) | 30.9 (8.9) | 0.9 | -- |
| mean time to LP (SD) days | 159.8 (214.6) | 148.3 (182.9) | 0.6 | -- |
| mean EDSS baseline (SD) | 1.2 (0.8) | 2.0 (1.1) | 0.2 | -- |
| No. of subjects with OB=0 at baseline | 1/5 | 1/19 | 0.3 | -- |
| mono/multi FS at onset (no.) | 3/2 | 15/4 | 0.6 | -- |
| MRI at onset (mean lesions, SD) | 1.0 (0.7) | 1.3 (0.6) | 0.4 | -- |
| No. of subjects with MRI=0 at baseline | 1/5 | 2/19 | 0.4 | -- |
| mean time to conversion (SD) months | NA | 19.6 (15.9) | NA | -- |
| mean time of follow up (SD) months | 28.4 (18.3) | 49.9 (21.2) | 0.06 | -- |
| *(average m/z)* 1337,561443 (SD) | 0,5156793396 (0,52035512431) | 0,1048738257 (0,17569022065) | 0.046 | ns |
| *(average m/z))* 1424,537736 (SD) | 0,0219907184 (0,03164014431) | 0,1068868887 (0,08583109195) | 0.032 | ns |
| *(average m/z)* 1848,5024 (SD) | 0,1409737902 (0,13692383959) | 0,0562591818 (0,12157103540) | 0.030 | ns |
| *(average m/z)* 2701,92928 (SD) | 0,0213920914 (0,04783417055) | ND | 0.050 | ns |
| *(average m/z)* 3016,126477 (SD) | ND | 0,1132717039 (0,24094126743) | 0.047 | ns |
| *(average m/z)* 3669,917046 (SD) | ND | 0,6994989128 (0,90612490332) | 0.047 | ns |
| *(average m/z)* 3987,586673 (SD) | 0,0129841992 (0,02903355204) | ND | 0.050 | ns |
| *(average m/z)* 7051,933833 (SD) | 0,3822056658 (0,10767061770) | 0,2308819225 (0,10767061770) | 0.050 | ns |

FDR= False Discovery Rate for multiple hypothesis correction [22].
